# Topological Langmuir-cyclotron wave

**DOI:** 10.1126/sciadv.add8041

**Published:** 2023-03-31

**Authors:** Hong Qin, Yichen Fu

**Affiliations:** Princeton Plasma Physics Laboratory and Department of Astrophysical Sciences, Princeton University, Princeton, NJ 08540, USA.

## Abstract

A theory is developed to describe the topological Langmuir-cyclotron wave (TLCW), a topological excitation in magnetized plasmas recently identified by numerical simulations. As a topological wave in a continuous medium, the TLCW propagates unidirectionally without scattering in complex boundaries and can be explored as an effective mechanism to energize particles. We show that, because momentum space in continuous media is contractible in general, the topology of the wave bundles is trivial over momentum space that contains no degeneracy points. This is in stark contrast to condensed matters with periodic lattice structures that impose nontrivial topology on momentum space. In continuous media without lattice structures, nontrivial topology of the eigenmode bundles manifests over phase space, and it is the nontrivial topology over phase space that underpins the topological excitations, such as the TLCW. It is shown that the TLCW can be faithfully modeled by a generic tilted Dirac cone in phase space, whose entire spectrum, including the spectral flow, is given.

## Teaser

Topology predicts a type of wave excitation in magnetized plasmas that could lead to an effective mechanism to energize particles.

## INTRODUCTION

Topological wave in continuous media is an active research topic for its practical importance. For example, it was discovered (*1*, *2*) that the well-known equatorial Kelvin wave, which can trigger an El Niño episode (*3*), is a topological wave in nature. Topological waves in cold magnetized plasmas have been recently studied (*4*, *5*). Through comprehensive numerical simulations, a topological surface excitation called topological Langmuir-cyclotron wave (TLCW) was identified (*5*, *6*). As a topological wave in a continuous medium, the TLCW has topological robustness, i.e., it is unidirectional and free of scattering and reflection. Thus, the TLCW excitation is expected to be experimentally observable and can be explored as an effective mechanism to drive current and particle flow.

In the present study, we develop a theoretical framework to describe the TLCW. For this purpose, it is necessary to first resolve a long-standing theoretical difficulty in the study of topological excitations in continuous media (*1*, *2*, *4*, *5*, *7*–*9*), which are defined as media without the discrete translation symmetry due to lattice structures. For continuous media, momentum (wave number) space is not periodic, and the wave number takes values in ℝ*^n^* (*n* = 1, 2, or 3). This is in stark contrast to condensed matters with lattice structures that impose nontrivial topology on momentum space. Because of this difficulty, the Chern number is not well defined for the eigenmode bundles in continuous media over momentum space. Furthermore, we show that, for continuous media, eigenmode bundles over a two-dimensional (2D) momentum space containing no degenerate points are topologically trivial due to the contractibility of the base manifold. We overcome the difficulty by uncovering the nontrivial topology in phase space and prove that it is the fundamental physics underpinning topological modes in continuous media. This structure is similar to what has been developed for quantum waves in molecular physics (*2*, *10*–*13*).

We rigorously prove that the TLCW is produced by the nontrivial topology over phase space at the Weyl point due to the Langmuir wave-cyclotron wave resonance using tools of algebraic topology and an index theorem for spectral flows formulated by Faure (*2*). Most of the techniques developed are applicable to general topological waves in continuous media as well. The key developments of the present study are summarized as follows.

(1) The TLCW is theoretically described as a spectral flow of the global Hamiltonian pseudo-differential operator (PDO) $\hat{H}$ for waves in an inhomogeneous magnetized plasma. For this problem, the semiclassical parameter of the Weyl quantization operator, which maps the bulk Hamiltonian symbol *H* to $\hat{H}$, is identified as the ratio between electron gyro-radius and the scale length of the inhomogeneity. We emphasize the important role of the semiclassical parameters and the necessity to identify them for topological waves in continuous media according to the nature of the physics under investigation.

(2) We formally construct the Hermitian eigenmode bundles of the bulk Hamiltonian symbol *H* and show that it is the topology of the eigenmode bundles over noncontractible, closed manifolds in phase space that determines the properties of spectral flows for continuous media. We emphasize that the topology of eigenmode bundles on momentum (wave number) space that contains no degeneracy points is trivial in continuous media because, unlike in condensed matters, momentum space in continuous media is contractible. The noncompactness of the momentum plane in continuous media is irrelevant, so is whether an integer or noninteger index can be designed. Without modification, the Atiyah-Patodi-Singer (APS) index theorem (*14*) proved for spectral flows over *S*^1^ is only applicable to matters with periodic momentum space, and Faure’s index theorem (*2*) for spectral flows over ℝ-valued wave numbers should be adopted for continuous media.

(3) A boundary isomorphism theorem (Theorem 2) is proved to facilitate the calculation of Chern numbers of eigenmode bundles over a 2D sphere in phase space. The theorem also defines a topological charge of an isolated Weyl point in phase space using a topological method, i.e., without using any connection.

(4) An analytical solution of the global Hamiltonian PDO of a generic tilted Dirac cone in phase space is found, which generalizes the previous result for a straight Dirac cone (*2*). The spectral flow index of a tilted phase space Dirac cone is calculated to be one, and the mode structure of the spectral flow is found to be a shifted Gaussian function.

(5) These tools are applied to prove the existence of the TLCW in magnetized plasmas with the spectral flow index being one. The Chern theorem (Theorem 12), instead of the Berry connection or any other connection, was used to calculate the Chern number. In addition, it is shown that the TLCW can be faithfully described by a tilted Dirac cone in phase space.

The paper is organized as follows. In the “Problem statement and general properties of TLCW” section, we pose the problem to be studied and describe the general properties of the TLCW identified by numerical simulations. The “Additional numerical evidence of TLCW” section presents additional numerical evidence and simulation results of the TLCW. The existence of TLCW as a spectral flow is proven in Results by studying the nontrivial topology of the plasma wave bundles over phase space. We construct a tilted Dirac cone model for the TLCW in the “An analytical model for TLCW by a tilted phase space Dirac cone” section, and the entire spectrum of the PDO of a generic tilted Dirac cone, including its spectral flow, is solved analytically. Discussion summarizes the main results of the present study. In Methods, the theoretical methods used to study the nontrivial topology of eigenmode bundles over phase space, i.e., the construction of Hermitian eigenmode bundles of waves in continuous media and associated algebraic topological tools, are provided.

### Problem statement and general properties of TLCW

We first pose the problem to be addressed in the present study, introduce the governing equations, set up the class of equilibrium plasmas that might admit the TLCW, and describe its general properties.

Consider a cold magnetized plasma with fixed ions. The equilibrium magnetic field ***B***_0_ = *B*_0_***e****_z_* is assumed to be constant. Because the plasma is cold, any density profile *n*(***r***) is an admissible equilibrium. Denote by *L* ∼ ∣*n*/∇*n*∣ the characteristic scale length of *n*(***r***). There is no equilibrium electrical field and electron flow velocity, i.e., ***E***_0_ = 0 and ***v***_0_ = 0. The linear dynamics of the system is described by the following equations for the perturbed electromagnetic field ***E*** and ***B***, and the perturbed electron flow ***v***

$$\partial_{t}v=-eE/m_{e}-\text{Ω}v\times e_{z}$$ (1)

$$\partial_{t}E=c\nabla \times B+4\pi env$$ (2)

$$\partial_{t}B=-c\nabla \times E$$ (3)

where Ω = *eB*_0_/*m*_e_*c* is the cyclotron frequency, *m*_e_ is the electron mass, and *e* > 0 is the elementary charge. We normalize ***v*** by $1/\sqrt{4\pi n(r)m_{e}}$, *t* by 1/Ω, ***r*** by *L*, and ∇ by 1/*L*. In the normalized variables, Eqs. 1 to 3 can be written as

$$i\partial_{t}\psi =\hat{H}\psi$$ (4)

$$\psi =\left(\begin{array}{c} v\\ E\\ B \end{array}\right)$$ (5)

$$\hat{H}(r,-i\eta \nabla )=\left(\begin{array}{ccc} ie_{z}\times & -i\omega_{p} & 0\\ i\omega_{p} & 0 & i\eta \nabla \times \\ 0 & -i\eta \nabla \times & 0 \end{array}\right)$$ (6)

where *i****e****_z_*× and *i*η ∇ × denote 3 × 3 antisymmetric matrices corresponding to ***e****_z_* and ∇, respectively. For a generic vector ***u*** = (*u_x_*, *u_y_*, *u_z_*) in ℝ^3^, the corresponding 3 × 3 antisymmetric matrix is defined as

$$u\times \equiv \left(\begin{array}{ccc} 0 & -u_{z} & u_{y}\\ u_{z} & 0 & -u_{x}\\ -u_{y} & u_{x} & 0 \end{array}\right)$$ (7)

In Eq. 6, $\omega_{p}(r)=\sqrt{4\pi n(r)e^{2}/m_{e}}/\text{Ω}$ is the local plasma frequency normalized by Ω, and η ≡ *c*/(*L*Ω) ∼ ρ*_e_*/*L* is a dimensionless parameter proportional to the ratio between electron gyro-radius and the scale length of *n*(***r***). Here, η is assumed to be small, i.e., η ≪ 1, and it plays the role of the semiclassical parameter for the Weyl quantization of this problem.

The Weyl quantization operator

$${\mathrm{Op}}_{\eta }:f\to \hat{f}={\mathrm{Op}}_{\eta }(f)$$ (8)

maps a function in phase space *f*(***r***, ***k***), called a symbol, to a PDO $\hat{f}$ on functions ψ(***r***) on the *n*-dimensional configuration space. The operator $\hat{f}={\mathrm{Op}}_{\eta }(f)$ is defined by

$$\hat{f}\psi (r)=\frac{1}{{\left(2\pi \eta \right)}^{n}}\int \;f\left(\frac{r+s}{2},k\right)\exp\left(\frac{ik\;\cdot (x-y)}{\eta }\right)\psi (s)dsdk$$ (9)

In particular, we have $\hat{k}=-i\eta \nabla$.

For the $\hat{H}$ given by Eq. 6, its preimage *H*, i.e., the symbol *H* satisfying $\hat{H}={\mathrm{Op}}_{\eta }(H)$, is

$$H(r,k)=\left(\begin{array}{ccc} {ie}_{z}\times & -i\omega_{p} & 0\\ i\omega_{p} & 0 & -k\times \\ 0 & k\times & 0 \end{array}\right)$$ (10)

In quantum theory, the semiclassical parameter is typically the Plank constant ℏ, and it is a crucial parameter in the index theorems for spectral flows (*2*, *14*) of PDOs. For the plasma waves in the present study, the semiclassical parameter is identified to be η ≡ *c*/(*L*Ω), which is the ratio between electron gyro-radius and the scale length of the equilibrium plasma. Notice that, in the PDO $\hat{H}(r,-i\eta \nabla )$, the differential operator ∇ is normalized by 1/*L*, but, in the symbol *H*(***r***, ***k***), the wave number ***k*** is normalized by Ω/*c*, due to the small semiclassical parameter η strategically placed in the Weyl quantization operator Op_η_. This structure between the PDO and the symbol is required for the application of the index theorems for spectral flows (*2*, *14*). In the study of other topological properties of classical media, such as electromagnetic materials (*7*, *8*, *15*, *16*), fluid systems (*1*, *2*, *17*–*24*), and magnetized plasmas (*4*–*6*, *9*, *25*–*29*), we believe that appropriate semiclassical parameters should also be carefully determined first on the basis of the specific nature of the problems under investigation.

The utility of the small semiclassical parameter η is that it enables a split of fast and slow dynamics for the full Hamiltonian PDO $\hat{H}(r,-i\eta \nabla )$ (*2*, *10*, *11*, *13*). Because η is the coefficient of the highest-order spatial derivatives in $\hat{H}(r,-i\eta \nabla )$, when η is small, the dynamics contains two components with a large scale separation. The slow scale dynamics, governed by the symbol *H*, describes the envelope of the fast scale dynamics, and the oscillation frequency of the fast scale dynamics is determined by the amplitude of the slow dynamics of the envelope,as in the familiar Wentzel-Kramers-Brillouin (WKB) analysis. It has been demonstrated in the context of molecular physics (*2*, *10*, *11*, *13*) that the topology of eigenmode bundles of the symbol is linked to the spectral flow properties of the PDO $\hat{H}(r,-i\eta \nabla )$. Specifically, the APS type of index theorem by Faure (*2*) was formulated in the limit of η → 0.

In plasma physics, the symbol *H*(***r***, ***k***) is called the local Hamiltonian of the system, but it is known as the bulk Hamiltonian in condensed matter physics. Thus, in the present context, the phrases “bulk modes” and “local modes” have the same meaning, referring to the spectrum determined by *H*(***r***, ***k***) locally at each ***r*** and each ***k*** separately. The spectrum of the PDO $\hat{H}(r,-i\eta \nabla )$ will be called global modes. The edge modes, including topological edge modes, refer to the global modes of $\hat{H}(r,-i\eta \nabla )$ whose mode structures are nonvanishing only in some narrow interface regions. In molecular physics, the symbol is known as the semiquantum Hamiltonian, its eigenmodes over parameter space are called “global modes,” and its eigenmodes over a sphere surrounding a degeneracy point are called “local modes.” It is unfortunate that the phrases “local modes,” “edge modes,” and “global” modes, defined in different branches of physics, have very different meanings.

The nonvanishing magnetic field breaks the time-reversal symmetry of the continuous system under investigation. For topological modes in condensed matters with lattice structures (discrete translation symmetry), the magnetic field can be included by the Peierls substitution (*30*).

**Fig. 1.**
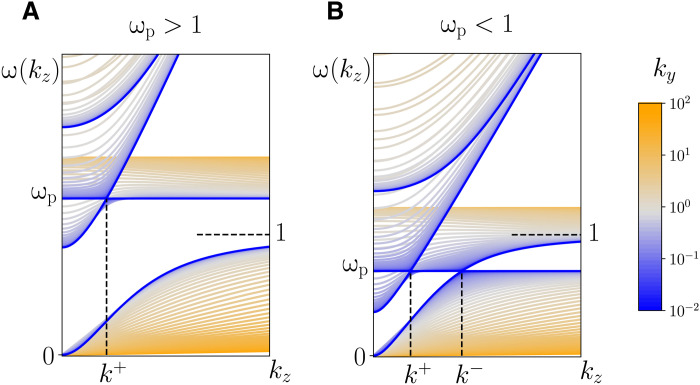
Dispersion relation of cold plasma waves. Plotted are the dispersion curves ω*_n_*(*k_z_*, *k_y_*) (*n* = 1, 2, 3, 4) for (**A**) an overdense plasma and (**B**) an underdense plasma. Different values of *k_y_* are indicated by the color map.

For a fixed ***r*** and a fixed ***k***, *H*(***r***, ***k***) is a 9 × 9 Hermitian matrix. Denote its 9 eigenmodes by

$$(\omega_{n},\psi_{n}),n=-4,-3,\ldots ,3,4$$

which are ordered by the value of the eigenfrequencies, i.e., ω*_i_* ≤ ω*_j_* for *i* < *j*. Under this index convention, it can be verified that ω_−*n*_ = −ω*_n_* and ω_0_ = 0, i.e., the spectrum is symmetric with respect to the real axis. Plotted in Fig. 1 are the dispersion relations of ω*_n_* (*n* = 1, 2, 3, 4) for an overdense and an underdense plasma, respectively. The eigenfrequencies are plotted as functions of *k_z_* and *k_y_* only because the spectrum is invariant when ***k*** rotates in the *xy* plane.

Straightforward analysis shows that, for a given ω_p_, the spectrum has two possible resonances, also known as (aka) Weyl points, when ***k***_⊥_ = 0 and *k_z_* = *k*^±^, where

$$k^{\pm }\equiv \frac{\omega_{p}}{\sqrt{1\pm \omega_{p}}}$$ (11)

are two critical wave numbers for the given ω_p_. We are interested in the resonance at ***k***_⊥_ = 0 and *k_z_* = *k*^−^, which is between the Langmuir wave and the cyclotron wave (the R-wave near the cyclotron frequency). Obviously, this Langmuir-cyclotron (LC) resonance or Weyl point exists when and only when the plasma is underdense, i.e., ω_p_ < 1.

For a given *k_z_*, the LC resonance occurs when ω_p_ = ω_pc_, where

$$\omega_{\mathrm{pc}}\equiv \frac{\sqrt{k_{z}^{4}+4k_{z}^{2}}-k_{z}^{2}}{2}$$ (12)

is the critical plasma frequency for the given *k_z_*. In the parameter space of (ω_p_, *k_x_*, *k_y_*) for a fixed *k_z_*, when moving away from the LC Weyl point (ω_p_, *k_x_*, *k_y_*) = (ω_pc_, 0, 0), the distance between ω_1_ and ω_2_ will increase, that is

$$\omega_{2}-\omega_{1}>0,\;\mathrm{when}\;(\omega_{p},k_{x},k_{y})\neq (\omega_{pc},0,0)$$ (13)

**Fig. 2.**
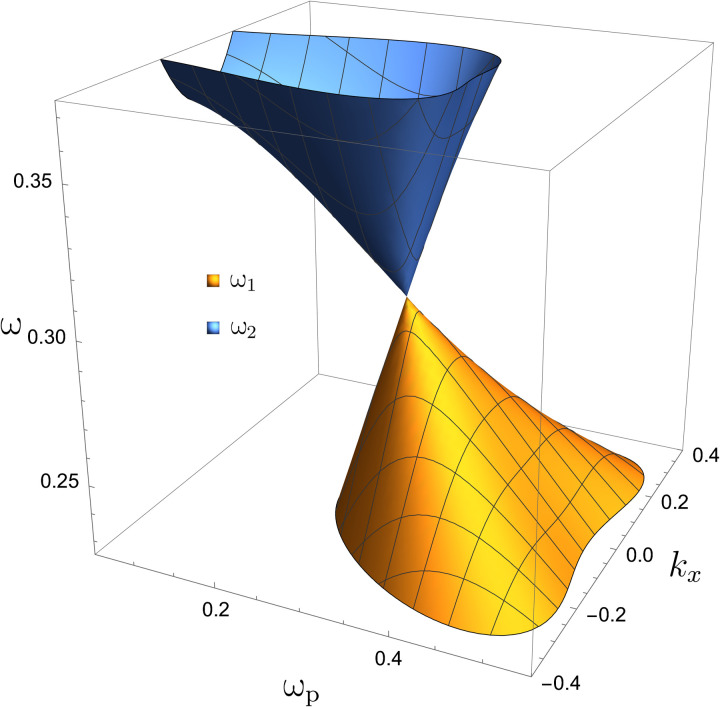
Tilted phase space Dirac cone. The surfaces of ω_1_ and ω_2_ form a tilted Dirac cone in the neighborhood of the LC Weyl point (ω_p_, *k_x_*, *k_y_*) = (ω_pc_, 0, 0).

Interesting topological physics happens in the neighborhood of the LC Weyl point (ω_p_, *k_x_*, *k_y_*) = (ω_pc_, 0, 0). Figure 2 shows the surfaces of ω_1_ and ω_2_ as functions of ω_p_ and *k_x_* near the LC Weyl point. The structure is known as a Dirac cone. One important feature of the Dirac cone at the LC Weyl point is that it is tilted. In addition, note that this tilted Dirac cone is in phase space because ω_p_ is a function of *x*. This is different from condensed matter physics, where the Dirac cone is mostly in momentum space.

Previous numerical studies and qualitative consideration (*5*, *6*) indicated that, for a given *k_z_*, a simple 1D equilibrium that is inhomogeneous in the *x* direction will admit the TLCW if the range of ω_p_(*x*) includes ω_pc_. In particular, we will consider the equilibrium profile that is homogeneous in region I (*x* ≤ −1) and region II (*x* ≥ 1), and ω_p_(*x*) monotonically decreases in the transition region (−1 ≤ *x* ≤ 1). The profile of ω_p_(*x*) satisfies the condition

$$\omega_{p1}>\omega_{p}(0)=\omega_{\mathrm{pc}}>\omega_{p2}$$ (14)

$$\omega_{p1}\equiv \omega_{p}(x\leq -1)$$ (15)

$$\omega_{p2}\equiv \omega_{p}(x\geq 1)$$ (16)

The LC Weyl point locates at *x* = 0, and ω_p1_ is the plasma frequency of region I and ω_p2_ that of region II. Note that, here, *x* is the dimensionless length normalized by *L*, the scale length of equilibrium density profile ω_p_(*x*). In classical magnetized plasmas, density profiles can always be modeled by functions with smooth derivatives. We assume the density profile does not have mathematical discontinuities. However, the derivatives can be arbitrarily large as long as they exist. For example, the method developed in the present analysis applies to a plasma-vacuum interface if the density is not strictly a Heaviside step function. In the following analysis, we will also assume that *k_z_* is a fixed parameter unless explicitly stated otherwise.

Through the variation of ω_p_(*x*), the spectrum ω*_n_*(*x*, *k_x_*, *k_y_*) of the bulk Hamiltonian symbol *H*(***r***, ***k***) becomes a function of *x*. We define the common gap condition for the spectra ω_1_(*x*, *k_x_*, *k_y_*) and ω_2_(*x*, *k_x_*, *k_y_*) as follows.

**Definition 1.** The spectra ω_1_(*x*, *k_x_*, *k_y_*) and ω_2_(*x*, *k_x_*, *k_y_*) are said to satisfy the common gap condition for parameters exterior to the ball $B_{r}^{3}\equiv \{(x,k_{x},k_{y})|x^{2}+k_{x}^{2}+k_{y}^{2}\leq r^{2}\}$ in the phase space of (*x*, *k_x_*, *k_y_*), if there exists an interval [*g*_1_(*r*), *g*_2_(*r*)] such that ω_1_(*x*, *k_x_*, *k_y_*) < *g*_1_(*r*) and ω_2_(*x*, *k_x_*, *k_y_*) > *g*_2_(*r*) for all $(x,k_{x},k_{y})\notin B_{r}^{3}.$ We call [*g*_1_(*r*), *g*_2_(*r*)] the common gap of ω_1_(*x*, *k_x_*, *k_y_*) and ω_2_(*x*, *k_x_*, *k_y_*) for parameters exterior to the ball $B_{r}^{3}$.

For all the parameter space that we have explored, condition 14 implies the common gap condition of ω_1_(*x*, *k_x_*, *k_y_*) and ω_2_(*x*, *k_x_*, *k_y_*) for (*x*, *k_x_*, *k_y_*) exterior to the ball of $B_{1}^{3}$. Because of the algebraic complexity of *H*(***r***, ***k***), this fact cannot be proved through a simple procedure, although no counter example was found numerically. In the “An analytical model for TLCW by a tilted phase space Dirac cone” section, we will give a proof of this fact for a reduced Hamiltonian corresponding to a tilted Dirac cone in the neighborhood of the LC Weyl point. In the analysis before the “An analytical model for TLCW by a tilted phase space Dirac cone” section, we will take the common gap condition as an assumption.

For the 1D equilibrium with inhomogeneity in the *x* direction, *k_y_* and *k_z_* are good quantum numbers and can be treated as system parameters. The PDO $\hat{H}(r,-i\eta \nabla )$ defined in Eq. 6 reduces to

$$\hat{H}(x,-i\eta \partial_{x},k_{y},k_{z})=\left[\begin{array}{ccc} ie_{z}\times & -i\omega_{p}(x) & 0\\ i\omega_{p}(x) & 0 & (i\eta \partial_{x},-k_{y,}-k_{z})\times \\ 0 & (-i\eta \partial_{x},k_{y,}k_{z})\times & 0 \end{array}\right]$$ (17)

and the corresponding bulk Hamiltonian symbol is

$$H(x,k_{x},k_{y},k_{z})=\left[\begin{array}{ccc} ie_{z}\times & -i\omega_{p}(x) & 0\\ i\omega_{p}(x) & 0 & (-k_{x},-k_{y,}-k_{z})\times \\ 0 & (k_{x},k_{y,}k_{z})\times & 0 \end{array}\right]$$ (18)

In region I or II, the system is homogeneous, and, in each region separately, it is valid to speak of the homogeneous eigenmodes of $\hat{H}(x,-i\eta \partial_{x},k_{y},k_{z})$, which are identical to the bulk modes of *H*(*x*, *k_x_*, *k_y_*, *k_z_*) in that region.

**Fig. 3.**
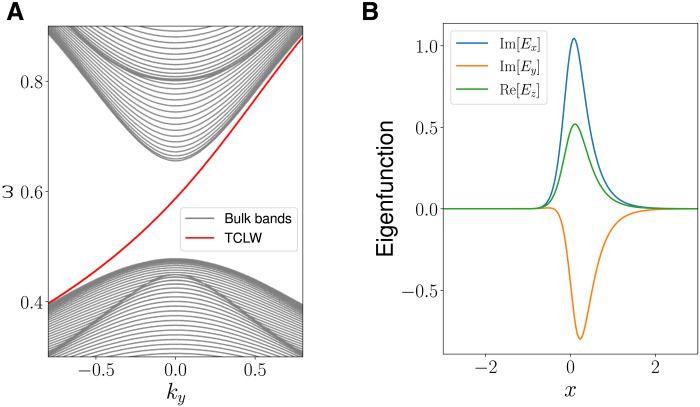
TLCW as a spectral flow. (**A**) Spectrum of $\hat{H}(x,-i\eta \partial_{x},k_{y},k_{z})$ as a function of *k_y_*. (**B**) The mode structure of the TLCW. The system parameters are (ω_p1_, ω_p2_, ω_pc_, *k_z_*) = (0.8, 0.45, 0.58, 0.9).

The TLCW is a global eigenmode of $\hat{H}(x,-i\eta \partial_{x},k_{y},k_{z})$ localized in the transition region of −1 < *x* < 1, hence the name of edge mode. In Fig. 3, the numerically calculated spectrum of $\hat{H}(x,-i\eta \partial_{x},k_{y},k_{z})$ is plotted as a function of *k_y_*. The system parameters are (ω_p1_, ω_p2_, ω_pc_, *k_z_*) = (0.8 ,0.45, 0.58, 0.9). The spectrum consists of three parts. The top and bottom parts are the spectrum of $\hat{H}(x,-i\eta \partial_{x},k_{y},k_{z})$ that fall in the bulk bands of *H*(*x*, *k_x_*, *k_y_*, *k_z_*) in regions I and II. The spectrum in the middle is a single curve passing through the common band gap shared by regions I and II. It is the TLCW. Its frequency increases monotonically with *k_y_*, passing through ω_pc_. Such a curve of the dispersion relation for the edge mode as a function of *k_y_* is known as spectral flow because it ships one eigenmode of $\hat{H}(x,-i\eta \partial_{x},k_{y},k_{z})$ from the bottom band to the top band across the band gap (see Fig. 3A). The frequency of the eigenmode in the gap can also decrease with *k_y_*, shipping one eigenmode of $\hat{H}(x,-i\eta \partial_{x},k_{y},k_{z})$ from the top band to the bottom band. We will call the first type upward edge mode and the second type downward edge mode. The spectral flow index is defined as the number of upward modes minus the number of downward modes. In Results, we will formally define spectral flow and show that the spectral flow index reflects the topology of the plasma waves and is determined by a topological index known as the Chern number of a closed manifold surrounding the Weyl point in the space of (*x*, *k_x_*, *k_y_*). This is why they are called topological edge modes. For the TLCW, we will show that its Chern number is 1. In the present analysis, frequency is normalized by the gyrofrequency Ω, and η scales as 1/Ω. When η decreases, the normalized frequency and spectrum density measured at *k_y_* = 0 are unchanged, but the scale length of topological edge mode decreases. This is expected because smaller η implies increased scale length separation between the fast and slow dynamics.

Condition 14 was identified as that for the existence of the TLCW (*5*, *6*) by heuristically applying the bulk-edge correspondence using the numerically integrated values of a regularized Berry curvature over the *k_x_*-*k_y_* plane for the bulk modes of *H*(*x*, *k_x_*, *k_y_*, *k_z_*) in regions I and II. However, such an integral should not be used as a topological index for the topology waves in continuous media, because the topology of vector bundles over a contractible base manifold is trivial, and the *k_x_*-*k_y_* plane that does not contain degeneracy points is contractible. A detailed discussion about the trivial topology over momentum space for continuous media can be found in the “Trivial topology of plasma waves in momentum space” section.

As explained above, the 1D inhomogeneous system for the TLCW is set up for a given *k_z_*, and, in the ℝ^3^ parameter space of (*x*, *k_x_*, *k_y_*), there is only one Weyl point located at (*x*, *k_x_*, *k_y_*) = (0, 0, 0). We can look at the topology of the eigenmode bundles over different 2D surfaces in the space of (*x*, *k_x_*, *k_y_*). Using the notion developed in molecular physics (*2*, *10*–*13*), an eigenmode bundle over a generic 2D surface is called an Λ-bundle, and the 2D surface as the base manifold of the Λ-bundle needs not to be closed (compact and without boundary). Many Λ-bundles are trivial, such as those over the *k_x_*-*k_y_* plane with *x* ≠ 0, and Chern numbers are not defined for Λ-bundles whose base manifolds are not closed. The Δ-bundles are the eigenmode bundles over the 2D spheres surrounding the Weyl point, and it turns out that the Δ-bundles encode nontrivial topological information linked to the spectral flow index of the PDO $\hat{H}(x,-i\eta \partial_{x},k_{y},k_{z})$. In the present study, the base manifolds of the Δ-bundles are surfaces that involve different values of *x*, *k_x_*, and *k_y_*. For this reason, we say that the Δ-bundles reside in the phase space. Here, the phrase “phase space” means a subset of phase space that cannot be defined as *x* = const. By Weyl quantization, the symbol *H*(*x*, *k_x_*, *k_y_*, *k_z_*) becomes an *x*-dependent operator in the *x* direction, $\hat{H}(x,-i\eta \partial_{x},k_{y},k_{z})$, but the argument *k_y_* remains a control parameter. This is similar to the systems studied in molecular physics (*2*, *10*–*13*).

In Results, we show how to formulate the bulk-edge correspondence for this problem in the continuous media, using an index theorem of spectral flow over ℝ-valued wave numbers established by Faure (*2*) and techniques of algebraic topology. We rigorously prove that there exists one TLCW when condition 14 and the common band gap condition are satisfied, after presenting additional numerical evidence of the TLCW in the next section. The Hermitian eigenmode bundle of plasma waves, with which the index theorem is concerned, is defined in Methods.

### Additional numerical evidence of TLCW

In this subsection, we display several more examples of numerically calculated TLCW by a 1D eigenmode solver of $\hat{H}(x,-i\eta \partial_{x},k_{y},k_{z})$ (*5*) and 3D time-dependent simulations (*6*).

**Fig. 4.**
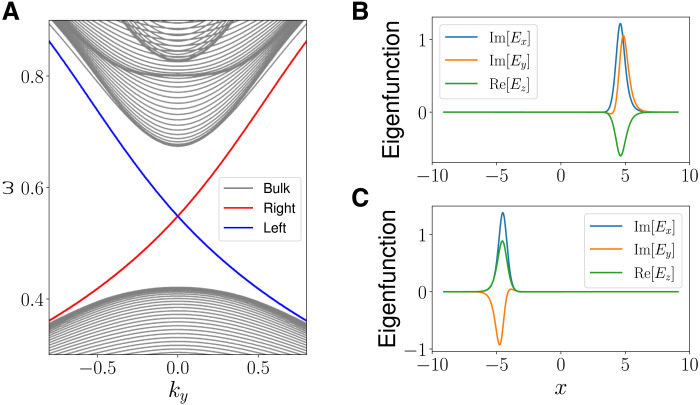
TLCWs at two boundaries. (**A**) Spectrum of $\hat{H}(x,-i\eta \partial_{x},k_{y},k_{z})$ as a function of *k_y_*. (**B**) The mode structure of the right TLCW. (**C**) The mode structure of the left TLCW. The system parameters are (ω_p1_, ω_p2_, ω_pc_, *k_z_*) = (0.8, 0.45, 0.58, 0.9).

The first example is the TLCW in a 1D equilibrium with the high-density region (ω_p1_ = 0.8) in the middle and the low-density region (ω_p2_ = 0.45) on the two sides. This equilibrium has two LC Weyl points at the two transitions between high-density and low-density regions. When condition 14 is satisfied, we expect to observe two TLCWs: one on the right LC Weyl point and one on the left. The numerically solved spectrum of $\hat{H}(x,-i\eta \partial_{x},k_{y},k_{z})$ is shown in Fig. 4, which meets the expectation satisfactorily.

**Fig. 5.**
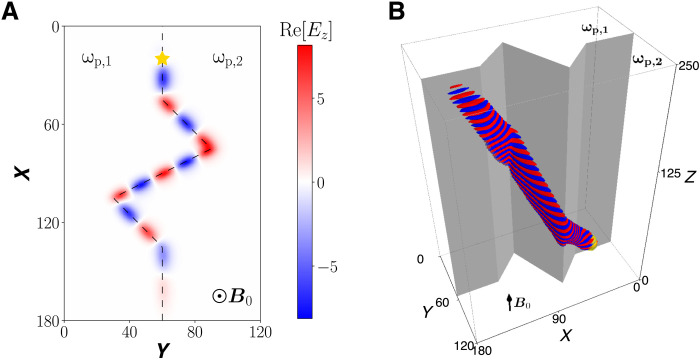
TLCW excited on a zig-zag boundary. (**A**) 2D simulation and (**B**) 3D simulation. The system parameters are (ω_p1_, ω_p2_, ω_pc_) = (0.8, 0.3, 0.54).

**Fig. 6.**
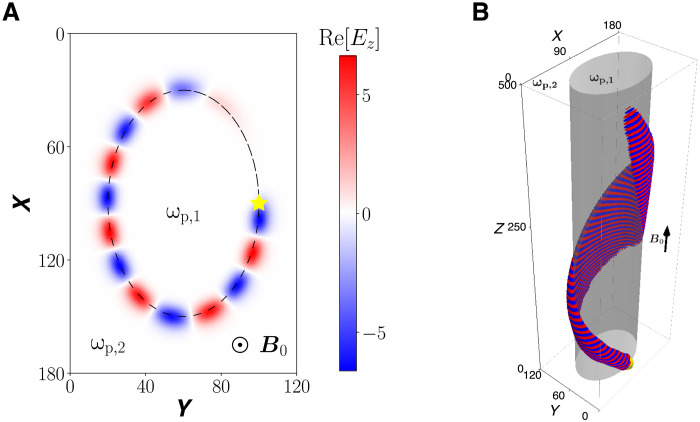
TLCW excited on an oval boundary. (**A**) 2D simulation and (**B**) 3D simulation. The system parameters are (ω_p1_, ω_p2_, ω_pc_) = (0.8, 0.3, 0.54). The TLCW propagates clockwise and carries an angular momentum.

Shown in Figs. 5 and 6 are 2D and 3D simulations of the TLCW, where the boundary between two regions are nontrivial curves in a 2D plane. In the simulations, an electromagnetic source is placed on the boundary marked by the yellow star. For the simulation in Fig. 5, the boundary is an irregular zigzag line. As anticipated, the TLCW propagates along the irregular boundary unidirectionally and without any scattering and reflection by the sharp turns. In Fig. 6, the boundary is a closed oval, and the TLCW stays on the oval boundary as expected. The propagation is again unidirectionally and without any scattering into other modes. What is demonstrated by the time-dependent 3D simulations in Figs. 5 and 6 is essentially the same TLCW eigenmode displayed in Fig. 3 in the 1D inhomogeneous system, if we view the transition boundaries in Figs. 5 and 6 as straight lines. This viewpoint is justified by the fact that the topological mode has a fast scale length across the boundaries and a slow scale length along the boundaries. The wave numbers along the boundaries manifested in Figs. 5 and 6 correspond to fixed values of *k_y_* in the spectral flow shown in Fig. 3. For comparison, the topological global effect discussed in molecular physics (*12*) is the formation of topologically coupled energy bands, and it can be attributed to the nontrivial second Chern classes. Because ω_p1_ > ω_p2_, the TLCW in Fig. 6 propagates clockwise and carries a nonzero (kinetic) angular momentum (*6*). Although the source does not carry any angular momentum, an angular momentum–carrying surface wave is generated by the mechanism of the TLCW.

## RESULTS

In this section, we rigorously prove the existence of TLCW by studying the nontrivial topology of plasma wave eigenmode bundles over phase space. The theoretical methods used, i.e., the construction of Hermitian eigenmode bundles of waves in continuous media and associated algebraic topological tools, are provided in Methods.

### Boundary isomorphism theorem

The following theorem is the main tool developed, which will enable us to analytically calculate the topological index for the TLCW over phase space. In a parameter space that is ℝ^3^, denote by $S_{r}^{2}=\{q=(q_{1,}\;q_{2,}\;q_{3})|q_{1}^{2}+q_{2}^{2}+q_{3}^{2}=r^{2}\}$ the sphere of radius *r* and by $Sh_{\left(a,b\right)}\equiv \{q=(q_{1,}q_{2,}q_{3})|a^{2}\leq q_{1}^{2}+q_{2}^{2}+q_{3}^{2}\leq b^{2}\}$ the 3D shell with inner radius *a* and outer radius *b*.

**Theorem 2.** (Boundary isomorphism) Let *E* → *Sh*_(*a*,*b*)_ be a Hermitian line bundle defined over a shell Sh_(*a*,*b*)_ in a parameter space that is ℝ^3^.

(i) The bundles obtained by restricting *E* over $S_{a}^{2}$ and $S_{b}^{2}$ are isomorphic, i.e., $E\to S_{a}^{2}≃E\to S_{b}^{2}$.

(ii) Bundles $E\to S_{a}^{2}$ and $E\to S_{b}^{2}$ have the same first Chern number, i.e., $n_{c}(E\to S_{a}^{2})=n_{c}(E\to S_{b}^{2})$.

Proof. To prove (i), construct the following continuous class of compressing maps on *Sh*_(*a*,*b*)_

$$\begin{array}{c} f_{\epsilon }:{\mathrm{Sh}}_{\left(a,b\right)}\to {\mathrm{Sh}}_{\left(a,b\right)}\\ p\mapsto \epsilon p+(1-\epsilon )\frac{ap}{|p|},0\leq \epsilon \leq 1 \end{array}$$ (19)

As ϵ decreases from 1 to 0 continuously, *f*_ϵ_ compresses the shell toward the inner sphere $S_{a}^{2}$. *f*_1_ is the identity map, *f*_0_ crashes the shell onto $S_{a}^{2},$ and *f*_1_ and *f*_0_ are homotopic. According to Theorem 16

$$f_{1}^{*}E≃f_{0}^{*}E$$ (20)

Restricting both sides of Eq. 20 to $S_{b}^{2}$ leads to

$$E|{}_{S_{b}^{2}}=(f_{1}^{*}E)|{}_{S_{b}^{2}}≃(f_{0}^{*}E)|{}_{S_{b}^{2}}=f_{0}^{*}(E_{S_{a}^{2}})|{}_{S_{b}^{2}}$$ (21)

Denote by *f*_0*r*_ the restriction of *f*_0_ on $S_{b}^{2}$, that is

$$\begin{array}{c} f_{0r}:S_{b}^{2}\to S_{a}^{2}\\ p\mapsto f_{0}(p) \end{array}$$ (22)

Obviously, *f*_0*r*_ is a diffeomorphism, and according to Theorem 17

$$f_{0}^{*}({E|}_{S_{a}^{2}})|{}_{S_{b}^{2}}=f_{0r}^{*}({E|}_{S_{a}^{2}})≃E|{}_{S_{a}^{2}}$$ (23)

Therefore

$$E\to S_{b}^{2}=E|{}_{S_{b}^{2}}≃E|{}_{S_{a}^{2}}=E\to S_{a}^{2}$$ (24)

For (ii), we have

$$f_{0r}^{*}[C_{1}(E\to S_{a}^{2})]=C_{1}[f_{0r}^{*}(E\to S_{a}^{2})]=C_{1}(E\to S_{b}^{2})$$

where the first equal sign is the naturality property of characteristic classes. The second equal sign is due to the fact that $f_{0r}^{*}(E\to S_{a}^{2})$ and $E\to S_{b}^{2}$ are two isomorphic bundles on $S_{b}^{2}$ and thus have the same Chern classes. The first Chern number on $E\to S_{b}^{2}$ is

$$\begin{array}{c} n_{c}(E\to S_{b}^{2})=\int_{S_{b}^{2}}C_{1}(E\to S_{b}^{2})\\ =\int_{S_{b}^{2}}f_{0r}^{*}[C_{1}(E\to S_{a}^{2})]\\ =\int_{S_{a}^{2}}C_{1}(E\to S_{a}^{2})=n_{c}(E\to S_{a}^{2}) \end{array}$$ (25)

where the integral on $S_{b}^{2}$ is evaluated on $S_{a}^{2}$ via the pullback mechanism in the third equal sign.

**Fig. 7.**
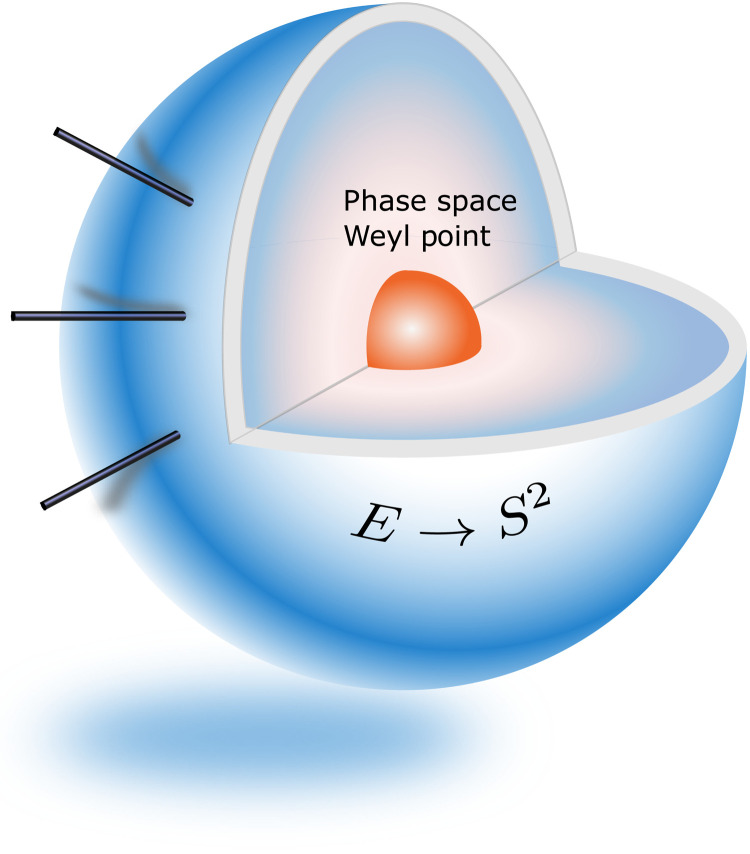
Topological charge of waves in continuous media. Around an isolated Weyl point, all closed surfaces surrounding the Weyl point support isomorphic eigenmode bundles and have the same first Chern number, which can be viewed as the topological charge associated with this isolated Weyl point in phase space for the isomorphic eigenmode bundles.

Theorem 2 says that, when the Hermitian line bundle *E* is defined on Sh(*a*, *b*), $E\to S_{a}^{2}$ and $E\to S_{b}^{2}$ are isomorphic bundles and have the same first Chern number. Note that identical Chern number is a necessary but not sufficient condition for bundle isomorphism. Two vector bundles over different base manifolds with the same Chern number could be totally unrelated. Around an isolated Weyl point, the Hermitian line bundle is well defined except at the Weyl point, Theorem 2 states that all closed surfaces surrounding the Weyl point support isomorphic eigenmode bundles and have the same first Chern number, which can be viewed as the topological charge associated with this isolated Weyl point in phase space for the isomorphic eigenmode bundles (see Fig. 7).

We also need the following results for the first Chern class for the plasma wave eigenmode bundles.

**Lemma 3.** For the nine bulk eigenmode bundles of the plasma waves specified by Hamiltonian symbol *H*(***r***, ***k***) defined in Eq. 10, the following identities for the first Chern class hold over a general base manifold without degeneracy points

$$C_{1}({⊕}_{j=1}^{4}E_{j})=C_{1}({⊕}_{j=-4}^{-1}E_{j})=0$$ (26)

$$C_{1}({⊕}_{j=-4}^{4}E_{j})=0$$ (27)

$$C_{1}(E_{0})=0$$ (28)

$$C_{1}({⊕}_{j=-4}^{1}E_{j})=C_{1}(E_{1})$$ (29)

They can be established straightforwardly from the fact the spectrum is symmetric with respect to the real axis. Note that Eq. 65 holds when the base manifold contains no degeneracy points.

### Faure’s index theorem for TLCW

In condensed matter physics, the bulk-edge correspondence states that the gap Chern number is equal to the number of edge modes in the gap. Mathematically, the correspondence had been rigorously proved as the APS index theorem (*14*) for spectral flows over *S*^1^, which corresponds to the momentum parameter *k_y_* in the direction with spatial translation symmetry of a periodic lattice. However, for waves in continuous media, including waves in plasmas, the *k_y_* parameter is not periodic, and it takes value in ℝ. Therefore, the APS index theorem proved for spectral flow over *S*^1^ is not applicable for waves in continuous media without modification. Recently, Faure (*2*) formulated an index theorem for spectral flows over ℝ-valued *k_y_*, which links the spectral flow index to the Chern numbers of the eigenmode bundles below the gap over a 2D sphere in the phase space of (*x*, *k_x_*, *k_y_*), which is known as a Δ-bundle. Faure’s index theorem applies to waves in continuous media. As discussed above, in a continuous medium, the eigenmode bundle over a *k_x_*-*k_y_* plane that contains no degeneracy point, also known as an Λ-bundle, is trivial, and its Chern number is not defined. In this and next subsections, we apply Faure’s index theorem and Theorem 2 to prove the existence of TLCW. For the bulk Hamiltonian symbol *H*(*x*, *k_x_*, *k_y_*, *k_z_*) defined in Eq. 18, the global Hamiltonian PDO $\hat{H}(x,-i\eta \partial_{x},k_{y},k_{z})$ defined in Eq. 17, and the 1D equilibrium profile specified by Eq. 14, we have the following theorems and definition adapted from Faure (*2*).

**Theorem 4.** For a fixed *k_z_*, assume that [*g*_1_, *g*_2_] is the common gap of ω_1_(*x*, *k_x_*, *k_y_*) and ω_2_(*x*, *k_x_*, *k_y_*) *f*or parameters exterior to the ball $B_{1}^{3}$. For any λ > 0, there exists η_0_ > 0 such that

(i) for all η < η_0_ and *k_y_* ∈ [−1 − λ,1 + λ], $\hat{H}(x,-i\eta \partial_{x},k_{y},k_{z})$ has no or discrete spectrum in the gap of [*g*_1_ + λ, *g*_2_ − λ] that depend on η and *k_y_* continuously;

(ii) for all η < η_0_, $\hat{H}(x,-i\eta \partial_{x},k_{y},k_{z})$ has no spectrum in [*g*_1_ − λ, *g*_2_ + λ] at *k_y_* = ± (1 + λ).

Proof. This theorem is a special case of theorem 2.2 in (*2*).

**Fig. 8.**
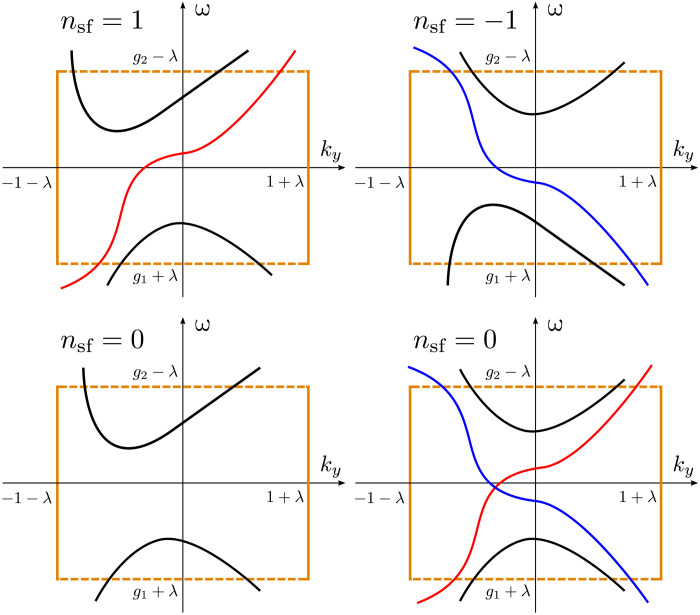
Illustration of possible spectral flows. Theorem 4 stipulates the traffic rules for the flow of the spectrum of $\hat{H}(x,-i\eta \partial_{x},k_{y},k_{z})$ in the common gap [*g*_1_ + λ, *g*_2_ − λ]. The index for red dispersion curves is 1, and the index for blue dispersion curves is −1.

Theorem 4 states that the spectrum of $\hat{H}(x,-i\eta \partial_{x},k_{y},k_{z})$ in the common gap [*g*_1_ + λ, *g*_2_ − λ], if any, must consist of discrete dispersion curves parameterized by *k_y_*. Theorem 4 also stipulates the following “traffic rules” for the flow of the spectrum. The dispersion curves cannot enter or exit the rectangle region [−1 − λ,1 + λ] × [*g*_1_ + λ, *g*_2_ − λ] on the *k_y_*-ω plane from the left or right side. They can only enter or exit through the top or bottom side (see Fig. 8). Intuitively, a spectral flow consists of dispersion curves of $\hat{H}(x,-i\eta \partial_{x},k_{y},k_{z})$ that can pass through the rectangle. It flows between the bottom band and the top band, as if transporting eigenmodes upward or downward through the spectral gap of *H*(*x*, *k_x_*, *k_y_*, *k_z_*) for parameters exterior to the ball $B_{1}^{3}$. We now formally define the spectral flow and spectral flow index.

**Definition 5.** For a fixed *k_z_*, assume that [*g*_1_, *g*_2_] is the common gap of ω_1_(*x*, *k_x_*, *k_y_*) and ω_2_(*x*, *k_x_*, *k_y_*) for parameters exterior to the ball $B_{1}^{3}$. An eigenmode in the gap is a smooth dispersion curve ω = *f*(*k_y_*, η) *of* $\hat{H}(x,-i\eta \partial_{x},k_{y},k_{z})$ satisfying the following condition: For any λ > 0, there exists an η_0_ > 0 such that either (i) for all η < η_0_, *f*(−1 − λ, η) < *g*_1_ + λ and *f*(1 + λ, η) > *g*_2_ − λ or (ii) for all η < η_0_, *f*(−1 − λ, η) > *g*_2_ − λ and *f*(1 + λ, η) < *g*_1_ + λ. For case (i), its index is 1. For case (ii), its index is −1. The spectral flow is the collection of all eigenmodes in the gap. The spectral flow index *n*_sf_ of $\hat{H}(x,-i\eta \partial_{x},k_{y},k_{z})$ is the summation of indices of all eigenmodes in the spectrum flow.

In the present context, the spectral flow of $\hat{H}(x,-i\eta \partial_{x},k_{y},k_{z})$ is the TLCW. However, Theorem 4 and Definition 5 are valid for any generic $\hat{H}$.

A few possible spectral flow configurations are illustrated in Fig. 8. Strictly speaking, *n*_sf_ is not necessarily the total number of all possible upward and downward eigenmodes in the spectral flow of $\hat{H}(x,-i\eta \partial_{x},k_{y},k_{z})$. It is the net number of upward eigenmodes in the spectral flow.

For plasma waves, the following theorem links the number of TLCWs to the first Chern number of the *E*_1_ eigenmode bundle over a noncontractible, closed surface in the phase space of (*x*, *k_x_*, *k_y_*).

**Theorem 6.** For a fixed *k_z_*, assume that the common gap condition for parameters exterior to the ball $B_{1}^{3}\equiv \{(x,k_{x},k_{y})|x^{2}+k_{x}^{2}+k_{y}^{2}\leq 1\}$ is satisfied for the spectra ω_1_(*x*, *k_x_*, *k_y_*, *k_z_*) and ω_2_(*x*, *k_x_*, *k_y_*, *k_z_*) of *H*(*x*, *k_x_*, *k_y_*, *k_z_*). The spectral flow index of $\hat{H}(x,-i\eta \partial_{x},k_{y},k_{z})$ in the gap is equal to the Chern number $n_{c}(E_{1}\to S_{1}^{2})$ of the *E*_1_ eigenmode bundle of *H*(*x*, *k_x_*, *k_y_*, *k_z_*) over $S_{1}^{2}\equiv \{(x,k_{x},k_{y})|x^{2}+k_{x}^{2}+k_{y}^{2}\leq 1\}$, i.e., $n_{\mathrm{sf}}=n_{c}(E_{1}\to S_{1}^{2})$.

Proof. This theorem is a direct specialization of theorem 2.7 formulated by Faure (*2*), which states that, when a spectral gap exists between ω*_l_* and ω_*l*+1_ of a bulk Hamiltonian for all parameters exteriors to $B_{r}^{3}$, the spectral flow index *n*_sf_ of the corresponding PDO in the gap is equal to the gap Chern number $n_{c}({⊕}_{j\leq l}E_{j}\to S_{r}^{2})$. For the plasma waves satisfying the common gap condition stated, *l* = 1 and

$$n_{\mathrm{sf}}=n_{c}({⊕}_{j\leq 1}E_{j}\to S_{1}^{2})=n_{c}(E_{1}\to S_{1}^{2})$$

where use is made of Lemma 3.

### Index calculation of TLCW using algebraic topological techniques

Theorem 6 links the number of TLCWs, or the spectral flow index of $\hat{H}(x,-i\eta \partial_{x},k_{y},k_{z})$, to the Chern number $n_{c}(E_{1}\to S_{1}^{2})$ of the *E*_1_ eigenmode bundle of *H*(*x*, *k_x_*, *k_y_*, *k_z_*) over $S_{1}^{2}\equiv \{(x,k_{x},k_{y})|x^{2}+k_{x}^{2}+k_{y}^{2}\leq 1\}$. However, it is not an easy task to calculate $n_{c}(E_{1}\to S_{1}^{2})$ either analytically or numerically. Here, we use the algebraic topological tools developed in the “Boundary isomorphism theorem” and “Nontrivial plasma wave topology in phase space” sections to analytically calculate $n_{c}(E_{1}\to S_{1}^{2}).$

Because, for the 1D equilibrium profile specified by Eq. 14, the LC Weyl point only occurs at *x* = 0 and the eigenmode bundle *E*_1_ is well defined in $B_{1}^{3}/(0,0,0)$, we can invoke Theorem 2 to calculate $n_{c}(E_{1}\to S_{1}^{2})$ as

$$n_{c}(E_{1}\to S_{1}^{2})=\lim_{\delta \to 0^{+}}n_{c}(E_{1}\to S_{\delta }^{2})$$ (30)

The right-hand side of Eq. 30 is the first Chern number of the *E*_1_ bundle over an infinitesimal sphere surrounding the Weyl point in the phase space of (*x*, *k_x_*, *k_y_*), and it can be analytically evaluated using Taylor expansion at the Weyl point as follows.

At the LC Weyl point (*x*, *k_x_*, *k_y_*) = (0, 0, 0), the spectrum and eigenmodes of *H*(*x*, *k_x_*, *k_y_*, *k_z_*) can be solved analytically. Denote by (ω_*j*0_, ψ_*j*0_) the *j*th eigenmode. At this point, two of the eigenmodes with positive frequencies resonate

$$\omega_{10}=\omega_{20}=\omega_{\mathrm{pc}}=\frac{\sqrt{k_{z}^{4}+4k_{z}^{2}}-k_{z}^{2}}{2}$$ (31)

and the corresponding eigenmodes are

$$\psi_{10}={\left(0,0,-\frac{i}{\sqrt{2}},0,0,\frac{1}{\sqrt{2}},0,0,0\right)}^{T}$$

$$\psi_{20}={\left(ik_{z},-k_{z},0,\frac{\omega_{\mathrm{pc}}}{k_{z}},i\frac{\omega_{\mathrm{pc}}}{k_{z}},0,-i,1,0\right)}^{T}$$

where ψ_10_ is the Langmuir wave and ψ_20_ is the cyclotron wave. In the infinitesimal neighborhood of the Weyl point, *k_x_* ∼ *k_y_* ∼ *x* ∼ δ

$$H(x,k_{x},k_{y},k_{z})=H_{0}+\delta H$$ (32)

$$H_{0}(x,k_{x},k_{y},k_{z})=\left[\begin{array}{ccc} ie_{z}\times & -i\omega_{\mathrm{pc}} & 0\\ i\omega_{\mathrm{pc}} & 0 & (0,0,-k_{z})\times \\ 0 & (0,0,k_{z})\times & 0 \end{array}\right]$$ (33)

$$\delta H(x,k_{x},k_{y},k_{z})=\left[\begin{array}{ccc} 0 & -i\omega_{p}^{\prime }(x)x & 0\\ i\omega_{p}^{\prime }(x)x & 0 & (-k_{x},-k_{y},0)\times \\ 0 & (k_{x},k_{y},0)\times & 0 \end{array}\right]$$ (34)

We can express *H* in the basis of ψ_*j*0_ (−4 ≤ *j* ≤ 4). However, for modes with δω = ω − ω_pc_ ∼ δ, *H* can be approximated by the expansion using ψ_10_ and ψ_20_ only and reduces to a 2 × 2 matrix

$$H_{2}(x,k_{x},k_{y},k_{z}):=\left(\begin{array}{cc} \psi_{10}^{†}H\psi_{10} & \psi_{10}^{†}H\psi_{20}\\ \psi_{20}^{†}H\psi_{10} & \psi_{20}^{†}H\psi_{20} \end{array}\right)=\left(\begin{array}{cc} \omega_{\mathrm{pc}}+\delta \omega_{p} & \frac{-k_{x}-ik_{y}}{\sqrt{2}\alpha }\\ \frac{-k_{x}+ik_{y}}{\sqrt{2}\alpha } & \omega_{pc}-\frac{4\omega_{\mathrm{pc}}}{\alpha^{2}}\delta \omega_{p} \end{array}\right)$$ (35)

$$\alpha \equiv \sqrt{4+3k_{z}^{2}-k_{z}\sqrt{4+k_{z}^{2}}},\delta \omega_{p}=-\beta x,\beta \equiv {|\frac{d\omega_{p}}{dx}|}_{x=0}\geq 0$$ (36)

where we have used the fact that the equilibrium profile ω_p_(*x*) selected in Eq. 14 decreases monotonically. The eigen system of *H*_2_ can be solved straightforwardly. The two eigenfrequencies of *H*_2_ are

$$\omega_{1}=\omega_{\mathrm{pc}}-\frac{\beta }{2}\left(1-\frac{4\omega_{\mathrm{pc}}}{\alpha^{2}}\right)x-\gamma$$ (37)

$$\omega_{2}=\omega_{\mathrm{pc}}-\frac{\beta }{2}\left(1-\frac{4\omega_{\mathrm{pc}}}{\alpha^{2}}\right)x+\gamma$$ (38)

$$\gamma \equiv \sqrt{\frac{k_{x}^{2}+k_{y}^{2}}{2\alpha^{2}}+\frac{x^{2}\beta^{2}}{4}{\left(1+\frac{4\omega_{\mathrm{pc}}}{\alpha^{2}}\right)}^{2}}$$ (39)

The corresponding eigenmodes, expressed in the basis of ψ_10_ and ψ_20_, are

$${\tilde{\psi }}_{1}={\left[\alpha \beta \left(1+\frac{4\omega_{\mathrm{pc}}}{\alpha^{2}}\right)x+2\alpha \gamma ,\sqrt{2}(k_{x}-ik_{y})\right]}^{T}$$ (40)

$${\tilde{\psi }}_{2}={\left[\alpha \beta \left(1+\frac{4\omega_{\mathrm{pc}}}{\alpha^{2}}\right)x-2\alpha \gamma ,\sqrt{2}(k_{x}-ik_{y})\right]}^{T}$$ (41)

Everywhere except (*x*, *k_x_*, *k_y_*) = (0, 0, 0) in the parameter space, we have ω_1_ < ω_2_, so the *E*_1_ eigenmode bundle of *H* is faithfully represented by ${\tilde{\psi }}_{1}$ when δ is small but nonvanishing. What matters for the present study is the first Chern number $n_{c}(E_{1}\to S_{\delta }^{2}),$ which can be obtained by counting the number of zeros of ${\tilde{\psi }}_{1}$ on $S_{\delta }^{2}$, according to Theorem 12.

On $S_{\delta }^{2}$, ${\tilde{\psi }}_{1}$ is well defined everywhere and has one zero at (*x*, *k_x_*, *k_y_*) = (−δ, 0, 0). The index of this zero can be calculated according to Definition 11 as follows. We select the following local frame for $E_{1}\to S_{\delta }^{2}$ in the neighborhood of (*x*, *k_x_*, *k_y_*) = (−δ, 0, 0),

$$e={\left[\frac{\alpha \beta (1+\frac{4\omega_{\mathrm{pc}}}{\alpha^{2}})x+2\alpha \gamma }{\left(k_{x}-ik_{y}\right)},\sqrt{2}\right]}^{T}$$ (42)

It is easy to verify that *e* is well defined in the neighborhood of (*x*, *k_x_*, *k_y_*) = (−δ, 0,0 ) on $S_{\delta }^{2}$, especially at the point of (*x*, *k_x_*, *k_y_*) = (−δ, 0, 0) itself. Note that *e* is singular at (*x*, *k_x_*, *k_y_*) = (δ, 0 ,0) on $S_{\delta }^{2}$; therefore, it is not a (global) section of bundle $E_{1}\to S_{\delta }^{2}$. The expression of the section ${\tilde{\psi }}_{1}$ in the *e* frame is (*k_x_* − i*k_y_*). In one turn on $S_{\delta }^{2}$ circulating (*x*, *k_x_*, *k_y_*) = (−δ, 0, 0), for example, on a circle with a fixed *x* near (*x*, *k_x_*, *k_y_*) = (−δ, 0, 0), the phase increase of (*k_x_* − *ik_y_*) is 2π. Thus, we conclude that Ind[(*x*, *k_x_*, *k_y_*) = (−δ, 0, 0)] = 1.

According to Theorem 12

$$n_{c}(E_{1}\to S_{\delta }^{2})=\mathrm{Ind}[(x,k_{x},k_{y})=(-\delta ,0,0)]=1$$

and from Eq. 30 and Theorem 6

$$n_{\mathrm{sf}}=n_{c}(E_{1}\to S_{1}^{2})=n_{c}(E_{1}\to S_{\delta }^{2})=1$$

We conclude that there is one net upward eigenmode in the spectral flow, i.e., the TLCW, if the common gap condition is satisfied. This is the main result of our study, and we summarize it in the following theorem.

**Theorem 7.** For a fixed *k_z_*, assume that the common gap condition for parameters exterior to the ball $B_{1}^{3}\equiv \{(x,k_{x},k_{y})|x^{2}+k_{x}^{2}+k_{y}^{2}\leq 1\}$ is satisfied for the spectra ω_1_(*x*, *k_x_*, *k_y_*, *k_z_*) and ω_2_(*x*, *k_x_*, *k_y_*, *k_z_*) of *H*(*x*, *k_x_*, *k_y_*, *k_z_*). The spectral flow index of $\hat{H}(x,-i\eta \partial_{x},k_{y},k_{z})$ in the gap *n*_sf_ = 1.

There is an analogy between the topology of magnetized plasmas and that of Weyl semimetals (*31*). The base manifolds of wave bundles in magnetized plasmas are 2D spheres in the phase space of (*x*, *k_x_*, *k_y_*). For Weyl semimetals, the base manifold is the space of (*k_x_*, *k_y_*), which is topologically a 2D torus due to the lattice structure (discrete translation symmetry). In both cases, the base manifolds are closed (compact and without boundary) manifolds, where Chern numbers, as topological indices, are defined. However, important differences exist. For magnetized plasmas, the 2D spheres are embedded in the 3D space of (*x*, *k_x_*, *k_y_*), and there are many different 2D spheres for a fixed *k_z_*. For Weyl semimetals, the 2D torus of (*k_x_*, *k_y_*) is intrinsic. It is not embedded in a large manifold, and there is only one such torus for fixed *k_z_* and *x*. While we would like to compare wave bundles defined over different 2D spheres in (*x*, *k_x_*, *k_y_*) for magnetized plasmas using Theorem 2, it is not meaningful to compare vector bundles over “two different tori” in (*k_x_*, *k_y_*) for fixed *k_z_* and *x* in Weyl semimetals.

### An analytical model for TLCW by a tilted phase space Dirac cone

**Fig. 9.**
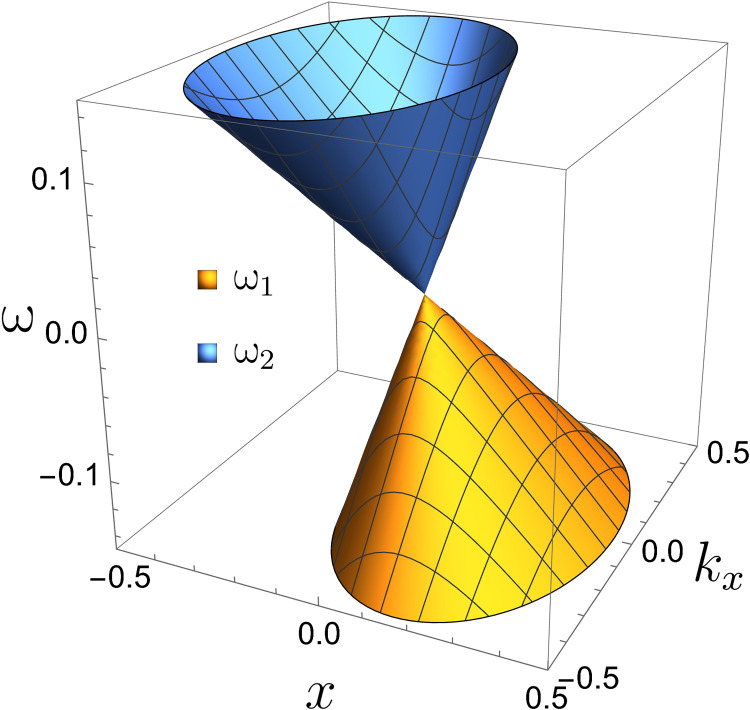
The tilted phase space Dirac cone of *H*_2_(*x, k_x_, k_y_, k_z_*). It faithfully represents the tilted Dirac cone of *H*(*x*, *k_x_*, *k_y_*, *k_z_*) at the LC Weyl point shown in Fig. 2.

As shown in the “Problem statement and general properties of TLCW” section, near the LC Weyl point, only the Langmuir wave and the cyclotron wave are important, and the 9 × 9 bulk Hamiltonian symbol *H*(*x*, *k_x_*, *k_y_*, *k_z_*) can be approximated by the 2 × 2 reduced bulk Hamiltonian symbol *H*_2_(*x*, *k_x_*, *k_y_*, *k_z_*). In the neighborhood of the LC Weyl point, *H*_2_(*x*, *k_x_*, *k_y_*, *k_z_*) and its PDO ${\hat{H}}_{2}(x,-i\eta \partial_{x},k_{y},k_{z})$ capture the physics of the TLCW. For the bulk modes of *H*(*x*, *k_x_*, *k_y_*, *k_z_*), the prominent feature near the LC Weyl point is the tilted Dirac cone shown in Fig. 2. This interesting structure is faithfully captured by *H*_2_(*x*, *k_x_*, *k_y_*, *k_z_*). For comparison, the tilted phase space Dirac cone of *H*_2_(*x*, *k_x_*, *k_y_*, *k_z_*) is plotted in Fig. 9. From the definition of *H*_2_(*x*, *k_x_*, *k_y_*, *k_z_*), it is clear that the factor 4ω_pc_/α^2^ is the reason for the cone being tilted.

For *H*_2_(*x*, *k_x_*, *k_y_*, *k_z_*), the corresponding PDO is

$${\hat{H}}_{2}(x,-i\eta \partial_{x},k_{y},k_{z})=\left[\begin{array}{cc} \omega_{\mathrm{pc}}-\beta x & \frac{i}{\sqrt{2}\alpha }(\eta \partial_{x}-k_{y})\\ \frac{i}{\sqrt{2}\alpha }(\eta \partial_{x}+k_{y}) & \omega_{\mathrm{pc}}+\frac{4\omega_{\mathrm{pc}}}{\alpha^{2}}\beta x \end{array}\right]$$ (43)

The result proved in previous sections shows that the full system $\hat{H}(x,-i\eta \partial_{x},k_{y},k_{z})$ admits one topological edge mode, i.e., the TLCW, as confirmed by numerical solutions in the “Problem statement and general properties of TLCW” and “Additional numerical evidence of TLCW” sections. This property of the TLCW is also faithfully captured by the reduced system ${\hat{H}}_{2}(x,k_{x},k_{y},k_{z})$.

In particular, Theorem 6 applies to ${\hat{H}}_{2}(x,-i\eta \partial_{x},k_{y},k_{z})$ as well, and the topology of eigenmode bundles of *H*_2_(*x*, *k_x_*, *k_y_*, *k_z_*) over the 2D sphere $S_{1}^{2}$ surrounding the LC Weyl point determines the spectral flow index of ${\hat{H}}_{2}(x,-i\eta \partial_{x},k_{y},k_{z})$. From Eqs. 37 and 38[Disp-formula E47], the common gap condition is satisfied for ω_1_ and ω_2_, and the proof of Theorem 6 shows that the Chern number $n_{c}(E_{1}\to S_{1}^{2})$ of the first eigenmode bundle of *H*_2_(*x*, *k_x_*, *k_y_*, *k_z_*) over $S_{1}^{2}$ is equal to 1. Thus, ${\hat{H}}_{2}(x,-i\eta \partial_{x},k_{y},k_{z})$ admits spectral flow with an index equal to one, which is the TLCW. In the present context, *k_z_* is fixed, and *k_y_* plays the role of a control parameter in the spectral flow. The Weyl point locates at (*x*, *k_x_*, *k_y_*) = (0, 0, 0). This situation can be compared with the rearrangement of quantum energy levels between bands in molecular systems (*10*, *11*, *13*).

To thoroughly understand the physics of a tilted phase space Dirac cone and the TLCW, we present here the analytical solution of the entire spectrum of ${\hat{H}}_{2}(x,k_{x},k_{y},k_{z})$, including its spectral flow in the band gap. For the PDO corresponding to a 2 × 2 symbol of a straight Dirac cone, its analytical solution has been given by Faure (*2*). However, for the PDO corresponding to a 2 × 2 symbol of a tilted Dirac cone, we are not aware of any previous analytical solution.

To analytically solve for its spectrum, we first simplify the matrix ${\hat{H}}_{2}(x,k_{x},k_{y},k_{z})$ in Eq. 43. Subtract the entire spectrum by ω_pc_ and renormalize *x* and *k_y_* as follows

$$\tilde{x}:=\sqrt{\frac{\sqrt{2}\alpha \beta \kappa }{\eta }}x,{\tilde{k}}_{y}:=\frac{k_{y}}{\sqrt{\sqrt{2}\alpha \beta \eta \kappa }}$$ (44)

where κ^2^ = 4ω_pc_/α^2^. Matrix ${\hat{H}}_{2}$ in Eq. 43 then simplifies to

$${\hat{H}}_{2}=\sqrt{\frac{\sqrt{2}\beta \eta \kappa }{\alpha }}\frac{1}{\sqrt{2}}\left[\begin{array}{cc} -\tilde{x}/\kappa & i(\partial_{\tilde{x}}-{\tilde{k}}_{y})\\ i(\partial_{\tilde{x}}+{\tilde{k}}_{y}) & \kappa \tilde{x} \end{array}\right]$$ (45)

It is clear that κ is a parameter measuring how tilted the Dirac cone is. When κ = 1, the Dirac cone is straight. However, for the TLCW, it is easy to confirm that κ > 1 for all possible system parameters.

From now on in this section, the overscript tilde in $\tilde{x}$ and ${\tilde{k}}_{y}$ will be omitted to simplify notation. We further transform ${\hat{H}}_{2}$ by a similarity transformation and scaling

$${{\hat{H}}_{2}}^{\prime }=\sqrt{\frac{\alpha }{\sqrt{2}\beta \eta \kappa }}R{\hat{H}}_{2}R^{-1}=\frac{1}{\sqrt{2}}\left[\begin{array}{cc} -x/\kappa & i(\partial_{x}-k_{y})/\kappa \\ i\kappa (\partial_{x}+k_{y}) & \kappa x \end{array}\right]$$ (46)

R=diag(κ,1) (47)

${{\hat{H}}_{2}}^{\prime }$ can be expressed using Pauli matrices and the identity matrix σ_0_ as

$$\sqrt{2}{{\hat{H}}_{2}}^{\prime }=i(\mu_{2}k_{y}+\mu_{1}\partial_{x})\sigma_{x}+(\mu_{1}k_{y}+\mu_{2}\partial_{x})\sigma_{y}-\mu_{1}x\sigma_{z}+\mu_{2}x\sigma_{0}$$ (48)

$$\mu_{1}=\frac{1}{2}\left(\kappa +\frac{1}{\kappa }\right),\mu_{2}=\frac{1}{2}\left(\kappa -\frac{1}{\kappa }\right)$$ (49)

We next apply a unitary transformation to cyclically rotate Pauli matrices such that (σ*_x_*, σ*_y_*, σ*_z_*, σ_0_) → (σ*_y_*, σ*_z_*, σ*_x_*, σ_0_). Under this rotation, ${{\hat{H}}_{2}}^{\prime }$ becomes

$${{\hat{H}}_{2}}^{\prime \prime }=\left(\begin{array}{cc} \mu_{1}\lambda +\mu_{2}\hat{a} & \mu_{2}\lambda -\mu_{1}{\hat{a}}^{†}\\ -\mu_{2}\lambda -\mu_{1}\hat{a} & -\mu_{1}\lambda +\mu_{2}{\hat{a}}^{†} \end{array}\right)$$ (50)

where $\lambda =k_{y}/\sqrt{2}$ and

$$\hat{a}=\frac{1}{\sqrt{2}}(x+\partial_{x}),{\hat{a}}^{†}=\frac{1}{\sqrt{2}}(x-\partial_{x})$$ (51)

are annihilation and creation operators. Notice that μ_1_ = 1 and μ_2_ = 0 when κ = 1, and this is the special case when ${{\hat{H}}_{2}}^{\prime \prime }$ reduces to a Hamiltonian corresponding to a straight Dirac cone (*2*, *32*). However, for the TLCW, κ > 1 holds everywhere. We now construct an analytical solution of ${{\hat{H}}_{2}}^{\prime \prime }$.

Recall that the eigenstates of a quantum harmonic oscillator ∣*n*〉 can be represented by the Hermite polynomials *H_n_*(*x*) as

$$\left\langle x|n\rangle =\varphi_{n}(x)=\frac{1}{{\left(2^{n}n!\sqrt{\pi }\right)}^{1/2}}e^{-\frac{x^{2}}{2}}H_{n}(x\right)$$ (52)

Define a set of shifted wave functions ∣*n*; δ〉 by

$$\left\langle x|n;\delta \rangle :=\varphi_{n}(x+\sqrt{2}\delta \right)$$ (53)

They satisfy the following iteration relations

$${\hat{a}}^{†}|n;\delta \rangle =\sqrt{n+1}|n+1;\delta \rangle -\delta |n;\delta \rangle$$ (54)

$$\hat{a}|n;\delta \rangle =\sqrt{n}|n-1;\delta \rangle -\delta |n;\delta \rangle$$ (55)

With these shifted wave functions as basis, it can be verified that ${{\hat{H}}_{2}}^{\prime \prime }$ has two sets of eigenvectors

$$\psi_{n}^{\pm }=\left(\begin{array}{c} |n+1;\delta_{n}^{\pm }\rangle \\ \gamma_{n}^{\pm }|n;\delta_{n}^{\pm }\rangle \end{array}\right),n=0,1,2,\ldots$$ (56)

where

$$\gamma_{n}^{\pm }=\frac{\sqrt{n+1}}{-\lambda \mp \sqrt{\lambda^{2}+n+1}},\delta_{n}^{\pm }=\pm \frac{\mu_{2}}{\mu_{1}}\sqrt{\lambda^{2}+n+1}$$

The corresponding eigenvalues are

$$E_{n}^{\pm }=\pm \frac{2\kappa }{1+\kappa^{2}}\sqrt{\lambda^{2}+n+1},n=0,1,2,\ldots$$ (57)

There is one additional eigenstate that is not included in Eq. 56, which represents the spectral flow. Its eigenvector and eigenvalue are

$$\psi_{-1}=\left(\begin{array}{c} |0;\delta_{-1}\rangle \\ 0 \end{array}\right),E_{-1}=\frac{2\kappa }{1+\kappa^{2}}\lambda$$ (58)

where

$$\delta_{-1}=\frac{\mu_{2}}{\mu_{1}}\lambda$$ (59)

Here, we abusively denote this eigenmode as the “*n* = −1” eigenstate. The spectral flow is a linear function of *k_y_*, and its mode structure is a shifted Gaussian function.

**Fig. 10.**
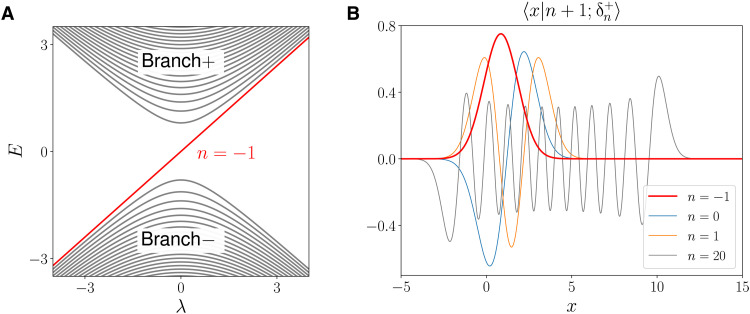
Analytical solutions of the tilted Dirac cone. (**A**) Analytical spectrum of ${{\hat{H}}_{2}}^{\prime \prime }(x,-i\partial_{x},\lambda )$ as a function of *k_y_*. (**B**) Analytical mode structure of the eigenmodes. The result of the TLCW (labeled by *n* = −1) agrees well with the numerical solution shown in Fig. 3.

The spectrum of ${{\hat{H}}_{2}}^{\prime \prime }(x,-i\partial_{x},\lambda )$ is plotted in Fig. 10A. The spectrum consists of three parts, and the top and bottom parts are the global modes in the frequency bands of *H*_2_(*x*, *k_x_*, *k_y_*, *k_z_*). The middle part is a single eigenmode in the spectral flow. Note that the tilted Dirac cone of *H*_2_(*x*, *k_x_*, *k_y_*, *k_z_*) breaks up into two pieces in the global modes of ${{\hat{H}}_{2}}^{\prime \prime }(x,-i\partial_{x},\lambda )$. In Fig. 10B, the analytical solutions of the mode structures of ${{\hat{H}}_{2}}^{\prime \prime }(x,-i\partial_{x},\lambda )$ are plotted. The TLCW in Fig. 10 is labeled by *n* = −1. In Fig. 10A, the spectrum flow moves from the bottom left to the top right across the gap between the bulk modes. The mode structure of the TLCW, as shown in Fig. 10B, is located in the transition region and has one peak, whereas those of the bulk modes extend beyond the transition region and have more than one peaks. The analytical result of the TLCW displayed in Fig. 10 agrees well with the numerical solution shown in Fig. 3.

## DISCUSSION

Inspired by advances in topological materials in condensed matter physics (*33*–*38*), the study of topological waves in continuous media, such as electromagnetic materials (*7*, *8*, *15*, *16*), fluid systems (*1*, *2*, *17*–*24*), and magnetized plasmas (*4*–*6*, *9*, *25*–*29*), has attracted much attention recently. The TLCW is a recently identified topological surface excitation in magnetized plasmas generated by the nontrivial topology at the Weyl point due to the Langmuir wave-cyclotron wave resonance (*5*, *6*). Here, we have systematically developed a theoretical framework to describe the TLCW.

It has been realized that the theoretical methodology for studying topological material properties in condensed matter physics cannot be directly applied to continuous media because the momentum (wave number) space for condensed matter is periodic, whereas that for continuous media is not. The typical momentum space for continuous media is ℝ*^n^* (*n* = 1, 2, 3), where Chern numbers are not defined. Specifically, the integral of the Berry curvature over *ℝ*^2^ is not the first Chern number. The difficulty has been attributed to the fact that* ℝ^n^* is not compact, and different remedies have been proposed accordingly. However, we demonstrated that the key issue is not whether momentum space is noncompact but, rather, that it is contractible. When the base manifold is contractible, all vector bundles on it are topologically trivial, and whether an integer index can be designed is irrelevant. For continuous media, eigenmode bundles are trivial over a 2D momentum space that contains no degeneracy points, and nontrivial topology manifests in vector bundles over phase space. Without modification, the APS index theorem (*14*) proved for spectral flows over *S*^1^ is only applicable to condensed matters, and Faure’s index theorem (*2*) for spectral flows over ℝ-valued *k_y_* should be adopted for continuous media.

In the present study, the TLCW is defined as the spectral flow of a PDO $\hat{H}$ for plasma waves in an inhomogeneous magnetized plasma, and the semiclassical parameter of the Weyl quantization operator is identified as the ratio between electron gyro-radius and the scale length of the inhomogeneity. We formally constructed the Hermitian eigenmode bundles of the bulk Hamiltonian symbol *H* corresponding to the PDO $\hat{H}$ and emphasized that the properties of spectral flows are determined by the topology of the eigenmode bundles over noncontractible, closed phase space manifolds. To calculate Chern numbers of eigenmode bundles over a 2D sphere in phase space, as required by Faure’s index theorem, a boundary isomorphism theorem (Theorem 2) was established.

The TLCW was proved to exist in magnetized plasmas as a spectral flow with the spectral index equal to 1. The Chern theorem (Theorem 12), instead of the Berry connection or any other connection, was used to calculate the Chern numbers. Last, we developed an analytically solvable model for the TLCW using a tilted phase space Dirac cone. An analytical solution of the PDO of a generic tilted phase space Dirac cone was found, which generalized the previous result for a straight Dirac cone (*2*). The spectral flow index of the tilted Dirac cone was calculated to be one, and the mode structure of the spectral flow was found to be a shifted Gaussian function.

As a topological edge wave, the TLCW can propagate unidirectionally and without reflection and scattering along complex boundaries. Because of this topological robustness, it might be relatively easy to excite the TLCW experimentally. Of course, laboratory and astrophysical plasmas are subject to many physical effects that have not been included in the present model, such as collisions and finite temperature. For practical application, these factors need to be carefully evaluated by experimental and theoretical methods. For example, the effect of disordered perturbation on topological modes can be modeled by theoretical methods based on stochastic Hamiltonians (*30*).

## METHODS

In this section, we define the Hermitian eigenmode bundles of waves in continuous media and developed associated algebraic topological tools used in the present study.

### Hermitian eigenmode bundles

The index theorem (*2*, *14*) establishes the bulk-edge correspondence linking the nontrivial topology of the bulk mode of symbol *H* and the spectral flow of PDO $\hat{H}$. The topology here refers to that of the Hermitian bundles of eigenmodes over appropriate regions of the parameter spaces, which we now define.

Denote the parameter space by *M*. In the present context, *M* is the space of all possible four tuples (*x*, *k_x_*, *k_z_*, *k_z_*). For a given *m* = (*x*, *k_x_*, *k_z_*, *k_z_*) ∈ *M*, the bulk Hamiltonian symbol *H*(*m*) supports a finite number of eigenmodes. For the plasma wave operator defined by Eq. 18, there are nine eigenmodes as explained in the “Problem statement and general properties of TLCW” section. However, most of the discussion and results in this section are not specific to the plasma waves and remain valid for a general bulk Hamiltonian symbol *H*(*m*) in continuous media with 1D inhomogeneity. When there is no degeneracy for a given eigenfrequency at a point *q* ∈ *M*, all eigenvectors corresponding to the eigenfrequency form a 1D complex vector space at *q*.

**Definition 8.** Let *Q* ⊂ *M* be a subset of the parameter space that forms a manifold with or without boundary. If the *j*th eigenmode is not degenerate over *Q*, then the space of disjointed union of all eigenvectors of ψ*_j_*(*q*) at all *q* ∈ *Q* forms a 1D complex line bundle

$$\pi_{j}:E_{j}\to Q$$ (60)

over *Q*. With the standard Hermitian form

$$\langle u,w\rangle \equiv u\;\cdot \bar{w}$$ (61)

for all $u,w\in \pi_{j}^{-1}(q)$ and *q* ∈ *Q*, *E_j_* → *Q* is also a Hermitian bundle. It will be called the Hermitian line bundle of the *j*th eigenmode of the bulk Hamiltonian symbol *H*(*m*) over *Q*.

If both the *l*th eigenmode and the *j*th eigenmode are nondegenerate over *Q*, the Whitney sum of *E_l_* → *Q* and *E_j_* → *Q* defines the Hermitian line bundle of the *l*th and *j*th eigenmodes

$$E_{\left\{l,j\right\}}\equiv E_{l}⊕E_{j}$$ (62)

with the Hermitian form defined as

$$\langle u,w\rangle \equiv u_{l}\;\cdot {\bar{w}}_{l}+u_{j}\;\cdot {\bar{w}}_{j}$$ (63)

for all ***u*** = (*u_l_*, *u_j_*), ***w*** = (*w_l_*, *w_j_*), where $u_{l},w_{l}\in \pi_{l}^{-1}(q)$ and $u_{j},w_{j}\in \pi_{j}^{-1}(q)$. Similarly, Hermitian bundle of a set of eigenmodes indexed by set *J* is defined as

$$E_{J}\equiv {⊕}_{j\in J}E_{j}$$ (64)

if for each *j* ∈ *J*, the *j*th eigenmode is not degenerate over *Q*. In general, *E_J_* can be defined when degeneracy exists only between indices in *J*, but we will not use this structure in the present study.

The current study is concerned with the topology of the Hermitian line bundles *E_j_* → *Q*. In particular, we would like to know when the bundle is trivial, i.e., a global product bundle over *Q*, and when it is not. If nontrivial, then it is desirable to calculate the Chern classes of the bundle to measure how twisted it is. For the Hermitian line bundles of eigenmodes of plasma waves, we show in Results that the topological index of the *E*_1_ bundle over a properly chosen noncontractible, closed manifold *Q* in phase space, calculated from its first Chern class *C*_1_(*E*_1_), determines the number of TLCWs at the transition region.

For Hermitian bundles, the associated principal bundles are *U*(*n*) bundles, and each Chern class *C_j_* is a de Rham cohomology class of the base manifold constructed from a curvature 2-form of the bundles. According to the Chern-Weil theorem, different connections for the bundles yield the same de Rham cohomology classes on the base manifold. In the present study, it is only necessary to calculate the first Chern class, and the following result is useful

$$C_{1}(E_{J})=\sum_{j\in J}C_{1}(E_{j})$$ (65)

The right-hand side of Eq. 65 is relatively easy to calculate because each *E_j_* is a Hermitian line bundle, whose first Chern class is given by

$$C_{1}=\frac{i}{2\pi }\theta$$ (66)

θ=dχ (67)

where θ is a curvature 2-form and χ is a connection. As mentioned above, different connections will generate the same *C*_1_ class. Nevertheless, the Hermitian line bundle is endowed with natural connection

χ=⟨w,dw⟩ (68)

which is a *u*(1)-valued local 1-form in each trivialization patch. This natural connection for the Hermitian line bundle is known as the Berry connection in condensed matter physics or the Simon connection. It was Barry Simon (*39*, *40*) who first pointed out that Berry’s phase is an anholonomy of the natural connection on a Hermitian line bundle. For this reason, Frankel (*41*) taunted the temptation to call it the Berry-Barry connection. However, as Simon pointed out, it is had been known to geometers such as Bott and Chern (*42*). Given the current culture of inclusion, it is probably more appropriate to call it the Bott-Chern-Berry-Simon connection.

### Trivial topology of plasma waves in momentum space

In terms of topological properties, there is a major difference between condensed matters and continuous media such as plasmas and fluids. Momentum space, or wave number space, of typical condensed matters is the Brillouin zone, which is noncontractible due to the periodicity of the lattices. On the contrary, the wave number space in plasmas and fluids is contractible, and it is a well-known fact that vector bundles over a contractible manifold are trivial. Here, a topological manifold *M* is called contractible if it is of the same homotopy type of a point, i.e., there exist a point *x*_0_ and continuous maps *f* : *M* → {*x*_0_} and *g* : {*x*_0_} → *M* such that *f* ∘ *g* is homotopic to identity in {*x*_0_} and *g* ∘ *f* is homotopic to identity in *M*. Because of its importance to the continuous media, we formalize this result as a theorem.

**Theorem 9.** Let *Q* be a subset of the parameter space *M* for a bulk Hamiltonian symbol *H*. If the *j*th eigenmode is nondegenerate on *Q* and *Q* is a contractible manifold, then the Hermitian line bundle *E_j_* → *Q* is trivial. In particular, the *n*th Chern class *C_n_*(*E_j_* → *Q*) = 0 for *n* ≥ 1.

Note that Theorem 9 holds for any bulk Hamiltonian symbol as long as the line bundle *E_j_* → *Q* is defined everywhere on *Q*, which implies that there is no degeneracy point on *Q*. If a contractible manifold *Q* contains a degeneracy point *p* and the line bundle is only defined over *Q*/{*p*}, which is not contractible in general, then the topology of *E_j_* → *Q*/{*p*} is nontrivial in general.

For the *H*(***r***, ***k***) defined in Eq. 10 and the *H*(*x*, *k_x_*, *k_y_*, *k_z_*) defined in Eq. 18 for plasma waves, a more specific result is available as a direct corollary of Theorem 9.

**Theorem 10.** For the bulk Hamiltonian symbol *H*(***r***, ***k***) defined in Eq. 10 for plasma waves, when *k_z_* ≠ *k*^±^, the Hermitian line bundle of all eigenmodes over the perpendicular wave number plane *Q*_*k*_⊥__ = {(*k_x_*, *k_y_*) ∣ *k_x_* ∈ ℝ, *k_y_* ∈ ℝ} = ℝ^2^ are trivial. In particular, *C_n_*(*E_j_* → *Q*_*k*_⊥__) = 0 for *n* ≥ 1 and −4 ≤ *j* ≤ 4.

The fact that *C_n_*(*E_j_* → *Q*) vanishes when *Q* is contractible is expected after all because *C_n_* is the de Rham cohomology class of the base manifold. However, Theorem 10 is important. It tells us that the plasma wave topology over the *k_x_*-*k_y_* plane is trivial if *k_z_* ≠ *k*^±^. Nontrivial topology of plasma wave bundles occurs only over noncontractible parameter manifolds, for example, over an *S*^2^ surface in the phase space of (*x*, *k_x_*, *k_y_*), as we will show in the “Nontrivial plasma wave topology in phase space” section.

Before leaving this subsection, we would like to point out that, in recent studies of wave topology in continuous media, much attention has been focused on the noninteger values of the integral of the Berry curvature (aka Simon curvature) over the 2D contractible *k_x_*-*k_y_* plane (ℝ^2^). In the present context, we will refer to the Berry curvature (aka Simon curvature) as curvature for simplicity. The most important fact in this regard is that the Chern theorem 12 is valid only for complex line bundles over a closed (compact and with boundary) base manifold. The Chern number is only defined for such a closed base manifold. The *k_x_*-*k_y_* space in a condensed matter with periodic lattice structure is a 2D torus and thus closed manifold, and the Chern theorem applies. However, the *k_x_*-*k_y_* space for a continuous media without periodic lattice structure is ℝ^2^, which is not a closed manifold, and the integral of the curvatures may not be an integer or even exist. Regardless of its values, such an integral does not reflect the topology of the eigenmode bundle and it should not be called a Chern number. As we see from Theorems 9 and 10, when the eigenmode bundle is well defined over the entire *k_x_*-*k_y_* plane, its topology is trivial due to the contractibility of ℝ^2^. It is not necessary or meaningful to design a topological index for it. Of course, we can still evaluate integrals of many possible bespoke curvatures over the 2D *k_x_*-*k_y_* plane and obtain the corresponding integer or noninteger values. However, these numbers are not Chern numbers, and they are not related to the topology of the eigenmode bundle that we already know to be trivial.

Researchers have proposed different curvature regularization techniques to select a different, modified curvature in the neighborhood of the curvature determined by the governing equations of the system such that the integral of the modified curvature is an integer. Some of the regularization techniques are argued to be motivated by specific physics-related considerations. Among all possible curvatures on the 2D *k_x_*-*k_y_* plane, some curvatures are probably more physically accurate than others. However, this does not change the fact that the topology of the eigenmode bundle over the 2D *k_x_*-*k_y_* plane is trivial, and the integral of chosen curvature is not a Chern number although it might be more physically accurate. An often-adopted regularization technique for magnetized plasmas is the short-wavelength cutoff method (*4*, *5*, *7*–*9*), which essentially modifies Newton’s law of motion by making the mass of classical charged particles increase with the wave number of the electromagnetic field in an ad hoc manner. It is difficult to argue this regularization results in more accurate physics in any sense, either classical or quantum-mechanical. It seems that its popularity is largely, if not entirely, based on the observation that its integral is an integer. Again, even if this regularization was more accurate in physics, it still would not change or be related to the fact that the eigenmode topology over the 2D *k_x_*-*k_y_* plane is trivial. The integral is an integer but not a Chern number.

### Nontrivial plasma wave topology in phase space

In the present context, the ultimate utility of the topological property of the eigenmode bundles of the bulk Hamiltonian symbol *H* is to predict the existence of the topological edge modes of the global Hamiltonian PDO $\hat{H}$. For this purpose, the proper plasma wave eigenmode bundles are over the 2D sphere in the parameter space of (*x*, *k_x_*, *k_y_*) for the *H*(*x*, *k_x_*, *k_y_*, *k_z_*) defined in Eq. 18

$$S_{1}^{2}=\{(x,k_{x},k_{y})|x^{2}+k_{x}^{2}+k_{y}^{2}=1\}=\partial B_{1}^{3}$$ (69)

where $B_{1}^{3}$ is the 3D ball with radius *r* = 1.

One indicator of nontrivial topology, or twist, of a wave eigenmode bundle over $S_{1}^{2}$ is the number of zeros a nontrivial section must have, akin to the situation of hairy ball theorem for the tangent bundle of *S*^2^. To include the possibilities of repeated zeros, we follow Frankel (*41*) to define the index of an isolated zero point *z* of a section *u* of a Hermitian line bundle as follows.

**Definition 11.** Let *z* be an isolated zero of a section *u* that has a finite number of zeros. Select a normalized local frame *e* for the Hermitian line bundle in the neighborhood of *z*. The normalized section near *z*, but not at *z*, can be expressed as *u*/∣*u*∣ = *e* exp (*i*α). The index at *z* is defined as

$$j_{u}(z)\equiv \frac{1}{2\pi }\lim_{D\to z}\int_{\partial D}\;d\alpha$$ (70)

where *D* is a small disk containing *z* on $S_{1}^{2}$ with orientation pointing away from $S_{1}^{2}$, and the orientation of ∂*D* is induced from that of *D*. The index of the section *u* is the sum of indices at all zeros *z_l_* of *u*

$$\mathrm{Ind}(u)\equiv \sum_{l}j_{u}(z_{l})$$ (71)

Note that, for each isolated zero *z*, the local frame *e* selected in the neighborhood of *z* is not vanishing at *z*. The index *j_u_*(*z*) intuitively measures how many turns the phase of *u* increases relative to *e* at *z* over one turn on *∂D*. In general, *e* is only a local frame instead of a global frame; otherwise, the bundle is trivial.

The following theorem of Chern relates the index of a nontrivial section to the first Chern class over *S*^2^ (*41*).

**Theorem 12.** (Chern) Let *E* be a Hermitian line bundle over a closed orientable 2D surface *S*^2^. Let *u* : *S*^2^ → *E* be a section of *E* with a finite number of zeros. Then, the integral of the first Chern class *C*_1_ over *S*^2^ is an integer that is equal to the index of the section *u*, that is

$$n_{c}\equiv \int_{S^{2}}C_{1}(E\to S^{2})=\mathrm{Ind}(u)$$

Here, *n*_c_ is known as the first Chern number. Because the present study only involves the first Chern number, it is denoted by *n*_c_ instead of *n*_c_1__. The first Chern number of the *j*th eigenmode bundle over $S_{1}^{2}$ is denoted by *n*_c*j*_. In Results, the first Chern number *n*_c1_ of the plasma wave eigenmode bundle *E*_1_ over $S_{1}^{2}$ is linked to the spectral flow index of $\hat{H}(x,-i\eta \partial_{x},k_{y},k_{z})$ using Faure’s index theorem (*2*). To facilitate the calculation of *n*_c*j*_ over $S_{1}^{2}$, we establish the following general facts about eigenmode bundles in continuous media, including plasma wave eigenmode bundles.

**Definition 13.** Let π_1_ : *E*_1_ → *P* and π_2_ : *E*_2_ → *Q* be two vector bundles. A diffeomorphism ϕ : *E*_1_ → *E*_2_ is called an isomorphism if, for every *p* ∈ *P*, ∃ *q* ∈ *Q* such that $\phi \circ \pi_{1}^{-1}(p)\subset \pi_{2}^{-1}(q)$ and $\phi :\pi_{1}^{-1}(p)\to \pi_{2}^{-1}(q)$ is a vector space isomorphism. If an isomorphism exists, then *E*_1_ and *E*_2_ are isomorphic, denoted as *E*_1_ ≃ *E*_2_.

Note that the base manifolds *P* and *Q* in the above definition can be identical or different.

**Definition 14.** Let *f* : *P* → *Q* be a smooth map between differential manifolds *P* and *Q*, and let π_2_ : *E* → *Q* be a vector bundle over *Q*. The pullback bundle π_1_ : f^*^E → *P* is defined as

$$f^{*}E=\{(p,e)\in P\times E|p\in P,e\in E,f(p)=\pi_{2}(e)\}$$ (72)

The standard manifold and vector bundle structure of f^*^E can be formally established. For example, see (*43*). Note that the pullback bundle is defined as a pullback set, and this mechanism does not define a map from *E* to f^*^E because *f* is not in general invertible. On the other hand, when *f* is invertible, a pullback map from *E* to f^*^E can be defined by a similar mechanism as follows.

**Definition 15.** Let *f* : *P* → *Q* be a diffeomorphism between differential manifolds *P* and *Q*, and let π_2_ : *E* → *Q* be a vector bundle over *Q*. The pullback map *f*^†^ is defined as

$$\begin{array}{c} f^{†}:E\to f^{*}E,\\ e\mapsto [f^{-1}\circ \pi_{2}(e),e] \end{array}$$ (73)

When *f* is a diffeomorphism, *f*^†^(*E*) = f^*^E.

We will use the following theorem known as homotopy induced isomorphism (*44*).

**Theorem 16.** (Homotopy induced isomorphism) Let *f*_0_ and *f*_1_ be the two homotopic maps between manifolds *P* and *Q.* For a vector bundle *E* → *Q*, the pullback bundles $f_{0}^{*}E$ and $f_{1}^{*}E$ over *P* are isomorphic.

Theorem 9 is a direct corollary of Theorem 16.

**Theorem 17.** Let *f* : *P* → *Q* be a diffeomorphism between differential manifolds *P* and *Q*, and let π_2_ : *E* → *Q* be a vector bundle over Q. The pullback map *f*^†^ is an isomorphism between *E* and f^*^E.

Proof. According to Definition 13, it suffices to prove that (i) *f*^†^ : *E* → f^*^E is a diffeomorphism and (ii) ∀*q* ∈ *Q*, ∃*p* ∈ *P* such that $f^{†}\circ \pi_{2}^{-1}(q)\subset \pi_{1}^{-1}(p)$ and $f^{†}:\pi_{2}^{-1}(q)\mapsto \pi_{1}^{-1}(p)$ is an isomorphism of vector space.

Because *f*^†^(*e*) = [*f*^−1^ ∘ π_2_(*e*), *e*] and both *f*^−1^ and π_2_ are smooth, *f*^†^ is smooth. By construction, *f*^†^ is smoothly invertible. Thus, *f*^†^ is a diffeomorphism. To prove (ii), we use a local trivialization. For ∀*q* ∈ *Q*, let *U* be an open set containing *q* in the open cover of *Q* for the local trivialization of *E* → *Q*. Locally, *E* is a production *U* × *V*. In particular, $\pi_{2}^{-1}(q)=\{q\}\times V$ and

$$f^{†}:(q,v)\mapsto (p=f^{-1}\circ \pi_{2}(q,v)=f^{-1}(q),(q,v))$$ (74)

For this fixed *q*

$$\begin{array}{c} f^{†}\circ \pi_{2}^{-1}(q)=f^{†}(\{(q,v)|v\in V\})=\{[p,(q,v)]|p=f^{-1}(q),v\in V\}\\ =\{[p,(q,v)]|f(p)=\pi_{2}(q,v),v\in V\}=\pi_{1}^{-1}(p) \end{array}$$ (75)

In addition, *f*^†^ : (*q*, *v*) ↦ [*p*, (*q*, *v*)] for the fixed *q* and *p* = *f*^−1^(*q*) is an isomorphism. Thus, *f*^†^ is an isomorphism and *E* ≃ f^*^E.
